# Communicating numeric quantities in context: implications for decision science and rationality claims

**DOI:** 10.3389/fpsyg.2015.00537

**Published:** 2015-04-30

**Authors:** David R. Mandel

**Affiliations:** Socio-Cognitive Systems Section, Defence Research and Development Canada and Department of Psychology, York UniversityToronto, ON, Canada

**Keywords:** decision-making, rationality, numeric quantifiers, context, linguistic interpretation

Perhaps more than most areas of cognitive psychology, the study of human judgment and decision-making relies heavily on experimental tasks communicated through written descriptions that convey numeric quantifiers as primary sources of information. Subjects in such studies usually are required to use such information to make choices, indicate preferences, or offer judgments. These responses are compared to normative benchmarks, resulting in the researchers drawing conclusions about the quality, coherence, or rationality of human judgment and decision-making (Arrow, 1982; Tversky and Kahneman, 1986; Stanovich and West, 2000). Most of this body of research has paid little attention (a) to how subjects interpret numeric information conveyed in writing and (b) to how those interpretations are influenced by context (Mandel and Vartanian, 2011; Teigen, in press). More often than not, researchers simply assume that subjects will interpret numeric quantities conveyed in experimental tasks as exact values, and also that subjects should interpret expressed numbers as precise quantities.

Yet it is uncontroversial in linguistics that numeric quantifiers may be treated as exact or approximate values, and where their interpretations are approximate, they may be treated as one-sided (e.g., *at least*, which is lower bounded, or *at most*, which is upper bounded) or two-sided (e.g., *roughly* or *about*). Linguistic accounts of numeric quantifiers (e.g., Horn, 1989; Carston, 1998; Levinson, 2000; Geurts, 2006; Breheny, 2008) do not support the normative claim (or assumption) that a precise “bilateral” reading of such quantifiers consistent with *exactly* is the proper reading. Although linguistic accounts differ in what they posit as possible semantic defaults, even those proposing a bilateral semantics, such as Breheny (2008), specify pathways for pragmatically derived unilateral interpretations, such as interpreting a numeric quantifier, *x*, as *at least x* or *at most x*.

More generally, the degree to which decision researchers seem confident in defining the meaning of linguistic terms for others runs counter to a fundamental idea in the philosophy of language, which holds that the meanings of words are definable only through their actual use in language (e.g., Wittgenstein, 1953; Austin, 1979). It also runs counter to psycholinguistic evidence indicating that even 5-year olds understand that numeric quantifiers should be interpreted as “at least” in some contexts (Musolino, 2004). And it runs counter to work in experimental pragmatics indicating that people develop context-sensitive scalar implicatures as they develop. For instance, they come to understand that although *some* logically entails *all*, it usually pragmatically excludes *all* because it would be infelicitous to use *some* if one meant *all* (Moxey and Sanford, 2000; Noveck, 2001; Noveck and Reboul, 2008).

## Studies of option framing as a case in point

Consider the following influential test of the coherence of decision-making:

Imagine that the U.S. is preparing for the outbreak of an unusual Asian disease, which is expected to kill 600 people. Two alternative programs to combat the disease have been proposed. Assume that the exact scientific estimates of the consequences of the programs are as follows:

[Positive Frame]

If Program A is adopted, 200 people will be saved.

If Program B is adopted, there is 1/3 probability that 600 people will be saved, and 2/3 probability that no people will be saved.

[Negative Frame]

If Program C is adopted, 400 people will die. If Program D is adopted, there is 1/3 probability that nobody will die, and 2/3 probability that 600 people will die.

According to Tversky and Kahneman (1981), options A and C in the Asian disease problem (ADP) are extensionally equivalent and likewise for options B and D. The former, moreover, are regarded by virtually all researchers who have used or commented on the problem as “certain” or “sure,” whereas the latter are regarded as “uncertain” or “risky.” Coherent choices thus require that a decision-maker who chooses A over B would also choose C over D (or vice versa).

Yet Tversky and Kahneman (1981) and others (e.g., see Levin et al., 1998, for an overview) found that most subjects choose A in the first pair and D in the second, ostensibly violating one of the most consensual normative principles of choice (Tversky and Kahneman, 1986)—description invariance, which states that extensionally equivalent events should not be differentially regarded merely because of the way in which they are described.

I say “ostensibly” because the claim that subjects presented with this problem violate description invariance (and, hence, are incoherent in their decision-making) rests on a shaky argument I call *proof by arithmetic*, which goes like this:

1. There are exactly 600 people at risk.
2. Option A will save exactly 200 people.
3. Option C will let exactly 400 people die.
4. Option A implies that exactly 400 people will die because exactly 600 minus exactly 200 is equal to exactly 400.
5. Option C implies that exactly 200 people will be saved because exactly 600 minus exactly 400 is equal to exactly 200.
6. Therefore, option A is equal to option C.

A similar argument can be expressed for the claim that options B and D are equivalent.

The reason the proof-by-arithmetic argument is shaky is that it assumes people interpret numeric quantifiers as exact values, when as noted earlier this reflects a naïve view on quantifiers, in particular, and language, in general.

To put that view to a proper test requires asking subjects not only about their choices but also about their interpretations of the quantifiers in the options presented to them. This approach was adopted in a recent experiment (Mandel, 2014, Experiment 3). After presenting subjects with a choice problem much like the ADP (except that it focused on 600 people at risk in a war-torn region rather than 600 people at risk due to an unusual Asian disease), they were asked whether they interpreted “200” in the positive frame or “400” in the negative frame as meaning (a) “at least [n],” (b) “exactly [n],” or (c) “at most [n].” Sixty-four percent responded “at least,” 30% responded “exactly,” and the remaining 6% responded “at most.”

This finding shows how untenable the proof-by-arithmetic argument is as a basis for the claim that subjects violate description invariance in framing problems like the ADP. Simply put, the researchers' interpretation was not shared by most subjects, who instead viewed the quantifiers presented to them as lower bounds. That result has profound consequences for the interpretation of subjects' choice data. Take the modal response: It is evident that saving at least 200 people [out of 600] is objectively better than letting die at least 400 people [out of 600]. Rather than being a “preference reversal” of dubious decision-making quality, for subjects who interpret the options as such, the pattern of choosing A over B and D over C may maximize subjective expected utility. In fact, when subjects' interpretations of the quantifiers in both options (i.e., A and B in the positive frame and C and D in the negative frame) were taken into account, a majority (76%) chose the option that was utility maximizing. Moreover, the framing effect reported by Tversky and Kahneman (1981) was found only in the subsample that reported a lower-bound interpretation of the quantifiers in options A or C. For those subjects who interpreted the quantifiers as exact values, there was no effect of frame.

Teigen and Nikolaisen (2009) also found evidence that numeric quantifiers are often interpreted as lower bounds. In one framing experiment that used a financial version of the ADP, subjects were asked which of two financial forecasters would be more accurate. Forecaster A predicted that NOK 250,000 (of 600,000) would be saved (in the positive frame) or that NOK 350,000 (of 600,000) would be lost (in the negative frame). Forecaster B predicted that NOK 150,000 (of 600,000) would be saved (in the positive frame) or that NOK 450,000 (of 600,000) would be lost (in the negative frame). In fact, NOK 200,000 was saved (NOK 400,000 was lost). In other words, the experiment was set up so that one forecaster overestimated the outcome and the other underestimated it, but they did so by the same amount (NOK 50,000). Supporting the hypothesis that people often spontaneously adopt a lower-bound interpretation of numeric quantifiers, the forecaster who overestimated the actual amount was judged to be more accurate. This was so regardless of whether the outcome entailed saving money or losing it (thus ruling out an alternative explanation based on desirability).

## Contexts matter

The preceding examples suffice to show that it is untenable for decision researchers to assume that subjects interpret numeric quantifiers as exact values. The next examples further demonstrate how aspects of context can moderate those interpretations. First, quantifier interpretations may be affected by the degree to which decision options are explicated. Consider the so-called certain options in the ADP: in A, nothing is said about the remaining 400 people; in C, nothing is said about the remaining 200. In contrast, the so-called uncertain options better (if not fully) resolve the uncertainty resulting from partial explication. That is, in options B and D the explicit probabilities add up to unity and for each possible outcome all 600 people in the focal set are accounted for—either they are all saved or else they all die. In this sense, the certain options seem less certain than the uncertain options. Mandel (2014, Experiment 3) resolved the uncertainty by filling in the missing information:

If Plan A is adopted, it is certain that 200 people will be saved and 400 people will not be saved.

If Plan C is adopted, it is certain that 400 people will die and 200 people will not die.

The effect of explicating the missing information on subjects' numeric quantifier interpretations was striking: only 24% selected “at least [n],” whereas 59% selected “exactly [n].” The remaining percentage of subjects who indicated “at most [n]” also nearly tripled (6 vs. 17%). The direction of these context-induced shifts is predictable: when all members of a focal set are referenced, it is likely that the speaker intends for the quantifiers to be exact. Thus, one might expect the bilateral interpretation to be modal, as was found. Yet one might also expect a smaller shift in favor of “at most,” which may reflect the reader's appreciation that the sum of the quantified subsets cannot exceed the value of the total set.

Moreover, the effect of sentential context (via the manipulation of explication) extends to choice: when both of the paired options were fully explicated, there was no effect of frame on subjects' choices (also see Kühberger, 1995; Mandel, 2001; Tombu and Mandel, 2015). Evidently, the interpretation of numeric quantifiers depends on aspects of sentential context, such as the explication of complementary implicit numeric quantifiers, and these context effects also affect subjects' choices. In this regard, the present discussion adds to a small literature that has highlighted the importance of context on decision-making (e.g., Wagenaar et al., 1988; Hilton, 1995; Goldstein and Weber, 1997; Rettinger and Hastie, 2001; Mandel and Vartanian, 2011).

Numeric quantifier interpretations are also affected by linguistic inferences that may be drawn from the broader semantic context of the decision-making problem. For instance, when a rationale for the values presented in the ADP was provided to subjects—namely, that there were only 200 vaccines for the disease that would be available—then a majority (71%) interpreted “200” as an upper bound (“at most”) in the positive frame, whereas a majority (64%) interpreted “400” as a lower bound (“at least”) in the negative frame (this experiment is reported in the General Discussion of Mandel, 2014). In contrast, when the standard ADP was presented, 58 and 54% gave the “at least” response in the positive and negative frames, respectively.

Once again, the direction of these interpretational shifts (both as a function of frame and whether a rationale was provided to subjects) is predictable, reflecting subjects' awareness that maximum quantities (i.e., having *only* 200 vaccines) set upper bounds on positive expected outcomes. And, once again, there is evidence that the effect of context on linguistic interpretation, in turn, influences the choices people make. When the vaccine rationale was provided in the ADP, no effect of frame on choice was found (Jou et al., 1996).

## Conclusion

William James wrote:

The great snare of the psychologist is the confusion of his own standpoint with that of the mental fact about which he is making his report. I shall hereafter call this the ‘psychologist’s fallacy' par excellence.” (1890/1950, p. 196, italics in original).

Decision researchers have perennially committed this fallacy by projecting their understanding of decision-task structure and meaning onto their subjects and then assessing the rationality of their subjects' judgments and choices *as if* their subjects invariably shared their views.

Over the years, a minority of psychologists have objected to that approach, having noted how subjects' task construals often differ from those assumed by experimenters (e.g., Henle, 1962; Berkeley and Humphreys, 1982; Phillips, 1983; Hilton, 1995). For instance, Gigerenzer (1996) stated:

Semantic inferences—how one infers the meaning of polysemous terms such as *probable* from the content of a sentence (or the broader context of communication) in practically no time—are extraordinarily intelligent processes. They are not reasoning fallacies.” (p. 593)

The research and arguments summarized here continue in a similarly critical vein, extending the alternative-task-construal argument to problems involving the linguistic interpretation of numeric quantifiers. As the examples provided here have illustrated, those linguistic interpretations are not only, at times, modally different from experimenters' interpretations, but also predictably moderated by multiple aspects of context. Such findings certainly do not prove that humans are rational, but they do show that some influential claims about human irrationality in decision-making are unwarranted. Such claims would benefit from careful consideration of possible linguistic effects on people's judgments and decisions.

### Conflict of interest statement

The author declares that the research was conducted in the absence of any commercial or financial relationships that could be construed as a potential conflict of interest.
